# Self-Concept Clarity and AI Anxiety in Graduate Students: Mediating Roles of Intentional Self-Regulation and Perceived Stress and Moderating Role of Intolerance of Uncertainty

**DOI:** 10.3390/bs16020171

**Published:** 2026-01-26

**Authors:** Qingqing Li, Yingmin Chen, Mingyang Zhang, Jia Zhang, Zhenrong Fu, Fei Ye

**Affiliations:** 1 Key Laboratory of Adolescent Cyberpsychology and Behavior, Ministry of Education, Central China Normal University, Wuhan 430079, China; liqing_psy@ccnu.edu.cn (Q.L.); chenym@mails.ccnu.edu.cn (Y.C.); zj0221@mails.ccnu.edu.cn (J.Z.); zrfu@ccnu.edu.cn (Z.F.); 2 Key Laboratory of Human Development and Mental Health of Hubei Province, School of Psychology, Central China Normal University, Wuhan 430079, China; 3 School of Law, Central China Normal University, Wuhan 430079, China; zhangmy_law@mails.ccnu.edu.cn; 4 Graduate School, Central China Normal University, Wuhan 430079, China

**Keywords:** self-concept clarity, intentional self-regulation, perceived stress, AI anxiety, intolerance of uncertainty

## Abstract

Research has identified self-concept clarity as a critical psychological resource; however, its mechanisms in mitigating artificial intelligence (AI) anxiety remain underexplored. This study employed a cross-sectional survey of 2176 graduate students (1584 females; *M_age_* = 23.60, *SD* = 2.03) to build a moderated chain mediation model that examines the mediating role of intentional self-regulation and perceived stress, as well as the moderating role of intolerance of uncertainty. Self-concept clarity was negatively correlated with AI anxiety, perceived stress, and intolerance of uncertainty, and positively correlated with intentional self-regulation. Mediation analyses showed that self-concept clarity predicted lower AI anxiety through both independent and chain mediation effects of intentional self-regulation and perceived stress. Moreover, intolerance of uncertainty moderated the links of self-concept clarity, intentional self-regulation, and perceived stress with AI anxiety. These findings highlight the importance and key explanatory mechanisms of self-concept clarity in mitigating AI anxiety among adults, elucidating that the cultivation of self-concept clarity and acceptance of uncertainty should be a crucial target for prevention and intervention strategies.

## 1. Introduction

In the era of artificial intelligence (AI), generative AI is rapidly integrating into and profoundly impacting higher education (Xia et al., 2024). On one hand, tools represented by ChatGPT (e.g., GPT-4) hold the potential to transform teaching and learning paradigms, for instance, by providing instant feedback and adaptive resources to support personalized learning (Asy’ari & Sharov, 2024). On the other hand, their widespread accessibility and powerful content-generation capabilities have raised a series of significant challenges. For example, students may over-rely on such tools to complete analytical and writing tasks that should be undertaken independently, thereby undermining their capacities for independent thinking, critical analysis, and creativity (Octaberlina et al., 2024; L. Zhang & Xu, 2024). Furthermore, students with low AI literacy may struggle to keep pace with the rapid iteration of technology, potentially intensifying feelings of knowledge inadequacy and fear of missing out. They may worry that falling behind in acquiring key skills could disadvantage them in future academic and professional competition (Polat, 2025; Yu, 2025), thereby contributing to heightened psychological stress. These challenges have given rise to a new psychological phenomenon known as AI anxiety. AI anxiety refers to the concern and anxiety about losing control over AI (Johnson & Verdicchio, 2017), which is not only related to students’ emotional reactions to technological progress, but also deeper concerns about their own value, competence, and future. As a vital force for academic innovation and intensive users of cutting-edge technology, graduate students face heightened pressure for academic output while also confronting the disruptive potential of generative AI in terms of skill displacement and ethical dilemmas (Kim et al., 2025). Consequently, their experience of AI anxiety is particularly salient and urgent. It is therefore crucial to investigate the protective factors and underlying mechanisms associated with AI anxiety.

Self-concept clarity refers to the extent to which a person’s self-concept is clearly defined, internally consistent, and temporally stable (Campbell et al., 1996). As a critical psychological resource, self-concept clarity has been linked to mental health outcomes, such as reduced loneliness, depression, and anxiety (Pang et al., 2024; Parise et al., 2019; Van Dijk et al., 2014), as well as adaptive behaviors, including lower risks of internet addiction and eating disorders (Vartanian et al., 2016; Y. Wang et al., 2022). However, there is a scarcity of research that directly examines whether and how self-concept clarity affects AI anxiety. Uncovering how self-concept clarity links to AI anxiety not only informs strategies to help people manage the threat perceptions and uncertainty triggered by AI technology, but also guides the development of evidence-based prevention and intervention strategies to reduce AI anxiety. To address these gaps, the current study aimed to investigate the association between self-concept clarity and AI anxiety among graduate students, while also exploring the potential mediating role of intentional self-regulation and perceived stress and the moderating role of intolerance of uncertainty.

### 1.1. Self-Concept Clarity and AI Anxiety

To the best of our knowledge, no research has explored the predicative effect of self-concept clarity on AI anxiety. However, extensive studies have confirmed a significant negative relationship between self-concept clarity and general anxiety (Kusec et al., 2016; Schwartz et al., 2012; Van Dijk et al., 2014). According to Crosby (1976), anxiety arises from perceived threats to one’s self-existence, often stemming from internal conflicts and directing towards unknown things in the future. Since self-concept serves as a fundamental framework for perceiving one’s own existence, its clarity may play a crucial role in alleviating anxiety.

Self-concept clarity is a structural component of self-concept, reflecting individual clarity, internal consistency, stability, and confidence in self-cognition (Campbell et al., 1996). The level of self-concept clarity directly affects an individual’s adaptability to the external environment and psychological resilience (Liang et al., 2022), thereby reducing vulnerability to anxiety (Hou et al., 2021; Stockinger et al., 2021). When individuals possess a high level of self-concept clarity, they have a clearer understanding of their abilities and worth (B. Zhang & Lin, 2023), which allows them to assess their roles and positions more precisely in the AI era, thereby reducing anxiety caused by AI-related challenges. In contrast, individuals with low self-concept clarity may feel confused and lost about their position in the world and future direction. When faced with a rapidly changing technological environment, they lack sufficient psychological resources to cope with potential threats, leading to heightened anxiety about their existence and the future (Lind & Van den Bos, 2002). Given that AI anxiety is a specific form of anxiety in AI-related situations and shares the core features and formation mechanisms of anxiety, this study hypothesizes that self-concept clarity negatively predicts AI anxiety.

### 1.2. The Mediating Role of Intentional Self-Regulation

Intentional self-regulation refers to a higher-order self-regulatory capacity that includes a series of goal-directed actions through which individuals proactively and consciously coordinate contextual demands, personal goals, and available resources to optimize developmental outcomes (Gestsdóttir & Lerner, 2008). Self-concept clarity not only enables individuals to better understand and accept themselves, but also helps them identify their needs and goals more accurately (Chen et al., 2024; Nezlek & Plesko, 2001). Self-regulation theory posits that self-concept plays a central role in the self-regulation process, and a clear and stable self-concept can promote effective self-regulation (Markus & Wurf, 1987). Conversely, uncertainty and confusion about oneself can disrupt the process of self-regulation and goal pursuit. Empirical evidence indicates that low self-concept clarity significantly inhibits self-control (Jiang et al., 2023), which is a critical component of intentional self-regulation (Light, 2017).

Individuals with high levels of intentional self-regulation tend to experience more positive emotions and adopt more effective coping strategies. Research shows that those with higher levels of intentional self-regulation are better equipped to choose more adaptive methods to reduce anxiety (Chang et al., 2020). The theory of selection, optimization, and compensation (SOC), one of the important theories of intentional self-regulation, proposes that individuals regulate their relationship with the environment through three strategies: selection, optimization, and compensation (Baltes & Baltes, 1990). The selection strategy involves individuals’ choices of goals and skills, such as choosing careers that are less susceptible to AI displacement or that leverage AI as a complementary tool (W. Li et al., 2024). The optimization strategy involves coordinating and allocating internal and external resources; optimizing skills and knowledge to better adapt to AI technology; mastering the basic operation of AI technology through training, learning, and practical application; and improving the understanding and application ability of AI (Shchepkina et al., 2024). Lastly, the compensation strategy involves developing alternative abilities such as soft skills, or finding new professional roles, to cope with the challenges posed by AI advancements (Babashahi et al., 2024). By employing these intentional self-regulation strategies, individuals can more effectively manage AI-related uncertainties and reduce AI anxiety. Therefore, intentional self-regulation may serve as a mediator between self-concept clarity and AI anxiety.

### 1.3. The Mediating Role of Perceived Stress

Perceived stress refers to an individual’s subjective feeling of distress when faced with threatening stimuli in the environment, often manifested as a sense of tension or loss of control (Xie & Fan, 2014). According to the identity disruption model of stress (Brown & McGill, 1989), a clear self-concept helps individuals cope with stress. Individuals with a clear and stable self-concept can identify and evaluate the sources of stress more accurately, enabling them to adopt adaptive coping strategies such as seeking social support, reassessing the situation, or taking direct action to solve problems, thereby reducing their sense of stress (Merdin-Uygur et al., 2018; Ritchie et al., 2011). Conversely, individuals with lower self-concept clarity often perceive the external world as chaotic and unpredictable, leading to heightened perceived stress (Smith et al., 1996). Recent longitudinal evidence further supported the negative relationship between self-concept clarity and perceived stress among a sample of young adults (Hertel et al., 2024).

Perceived stress is a well-established predictor of depression and anxiety (Shin & Park, 2024). In the context of AI-related challenges, individuals’ perceived threats posed by AI, such as job displacement and privacy risks, may enhance their perceived stress, thereby triggering anxiety (Xu et al., 2023). These stress-induced anxieties may manifest as worries about the future, distrust in AI technology, and doubts about their ability to remain competitive in the AI era (Glikson & Woolley, 2020). Given these findings, perceived stress is expected to play mediating role in the relationship between self-concept clarity and AI anxiety.

### 1.4. The Chain Mediation of Intentional Self-Regulation and Perceived Stress

Conservation of resources theory (Hobfoll, 1989) posits that individuals assess and respond to stress based on their available resources, which influence their ability to cope with challenging situations (Hobfoll et al., 2018). Those with high levels of intentional self-regulation are able to buffer stress by utilizing multidimensional assets, such as accumulating social capital, nurturing psychological capital, and restructuring time resources (Chang et al., 2020; Häfner et al., 2015; Siu, 2013). As a stress coping strategy, intentional self-regulation also fosters adaptive attitudes and behaviors toward AI technology. Individuals with higher intentional self-regulation are more likely to actively frame their perspectives and change their behavioral patterns to embrace AI technology, learning how to coexist and collaborate with it rather than perceiving it as a threat (Mahdi & Sahari, 2024; Trotter et al., 2023). This adaptive adjustment helps reduce perceived stress associated with technological changes (Pirkkalainen et al., 2019). Therefore, intentional self-regulation and perceived stress may function as a chain mediating process in the relationship between self-concept clarity and AI anxiety.

### 1.5. The Moderating Role of Intolerance of Uncertainty

Extensive research demonstrates that intolerance of uncertainty plays a crucial role in the development and persistence of anxiety and serves as a risk factor for generalized anxiety disorder (Andrews et al., 2023). Intolerance of uncertainty refers to the cognitive bias exhibited by individuals when perceiving, interpreting, and reacting to ambiguous or uncertain situations or events (Dugas et al., 2004). Intolerance of uncertainty is characterized as a cognitive vulnerability, with individuals prone to perceiving uncertain future events as threats (Koerner & Dugas, 2008). Additionally, research indicates that intolerance of uncertainty is linked to cultural values. Eastern cultures, which emphasize collectivism and long-term orientation, tend to exhibit greater adaptability and tolerance toward uncertainty. In contrast, Western cultures, inclined to seek absolute truth and emphasize certainty, often display stronger aversion to uncertainty (Guo et al., 2022; Hofstede, 1996). In this study, we primarily examine intolerance of uncertainty from an individual difference perspective, while acknowledging its cultural rootedness. In the context of AI-related concerns, research has demonstrated that intolerance of uncertainty not only influences individuals’ acceptance of AI technology but may also exacerbate anxiety stemming from the uncertainties associated with AI (Johnson & Verdicchio, 2017; Schiavo et al., 2024). Individuals with high levels of intolerance of uncertainty may experience more intense anxious reactions when confronted with AI, due to their difficulty in tolerating the uncertainties related to it. These uncertainties may arise from the “black box” nature of AI, which pertains to the opacity of its decision-making processes, concerns about societal transformations induced by AI, and ambiguities concerning AI’s ethical and liability aspects (De Freitas et al., 2023). This indicates that an individual’s tolerance for uncertainty may moderate the relationship between other psychological factors and AI anxiety. Therefore, examining the moderating role of intolerance of uncertainty can help clarify the boundary conditions underlying the formation mechanism of AI anxiety, thereby providing a basis for differentiated interventions. Accordingly, this study hypothesizes that intolerance of uncertainty moderates the relationship among self-concept clarity, intentional self-regulation, perceived stress, and AI anxiety.

### 1.6. The Current Study

**Figure 1:**
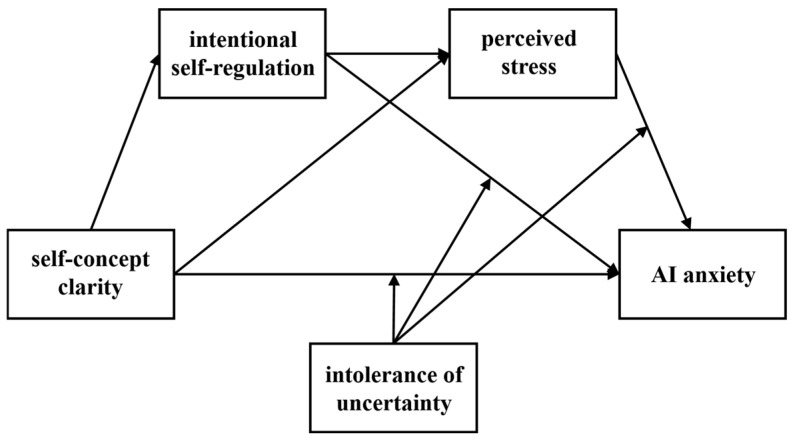
The proposed hypothesis model.

Although previous research has demonstrated a close relationship between self-concept clarity and mental health, no research has directly examined the predictive effect of self-concept clarity on AI anxiety. Furthermore, the psychological mechanisms underlying this relationship remain unclear. To address these gaps, the present study investigated the potential mechanistic pathways from self-concept clarity to AI anxiety among Chinese graduate students. Based on the above literature review, we constructed a moderated chain mediation model (see Figure 1) to understand the mechanism underlying this relationship. It was hypothesized that (1) self-concept clarity would negatively predict AI anxiety; (2) intentional self-regulation and perceived stress would independently mediate the relationship between self-concept clarity and AI anxiety; (3) intentional self-regulation and perceived stress would play a chain mediating role in the relationship between self-concept clarity and AI anxiety; and (4) intolerance of uncertainty would moderate the associations of self-concept clarity, intentional self-regulation, and perceived stress with AI anxiety.

## 2. Materials and Methods

### 2.1. Participants

This study conducted a cross-sectional online survey targeting master’s and doctoral students from multiple universities in central China. A total of 2380 responses were initially collected for the study. Responses were considered invalid if participants failed attention checks or engaged in straight-lining. After excluding 204 invalid questionnaires, a total of 2176 (ages 21 to 32 years, *M_age_* = 23.60, *SD* = 2.03) valid questionnaires were collected, resulting in an effective rate of 91.43%. Among the participants, there were 592 males (27.21%) and 1584 females (72.79%); 1321 (60.7%) were from urban areas and 855 (39.3%) were from rural areas. All participants were informed of the study objectives, and informed consent was obtained prior to participation. Ethical approval of this study was granted by the Ethics Committee of the University (CCNU-IRB-202404018A).

### 2.2. Measures

#### 2.2.1. Self-Concept Clarity

This study adopted the Chinese version of Self-Concept Clarity Scale (SCCS; Niu et al., 2016), originally developed by Campbell et al. (1996). The scale consists of 12 items (e.g., “In general, I have a clear sense of who I am and what I am”), using a 7-point Likert-type scale ranging from 1 (“strongly disagree”) to 7 (“strongly agree”). Ten items were reverse-scored such that higher average scores indicate higher levels of self-concept clarity. The validity and reliability of the Chinese SCCS have been confirmed in Chinese adolescents and adults (Xiang et al., 2022). In the current study, the Cronbach’s α coefficient of the scale is 0.88. Confirmatory factor analysis indicated good structural validity (CFI = 0.960, TLI = 0.949, RMSEA = 0.068).

#### 2.2.2. Intentional Self-Regulation

This study employed the Intentional Self-Regulation Scale, originally developed by Gestsdóttir and Lerner (2007), and translated and adapted by Dai et al. (2010). The scale contains 9 items that assess three dimensions: selection (3 items, e.g., “When I decide upon a goal, I stick to it”), optimization (3 items, e.g., “To achieve my goals, I will carefully think about the best way to complete my plan”), and compensation (3 items, e.g., “When I encounter something I cannot solve, I will ask others to help me”). All items were rated on a 5-point scale from 1 (“completely disagree”) to 5 (“completely agree”). The average scores of all items were calculated, with higher scores indicating better intentional self-regulation ability. The scale was validated in Chinese youth with good reliability and validity (Gan et al., 2022). In this study, the Cronbach’s α coefficient is 0.89. Confirmatory factor analysis indicated good structural validity (CFI = 0.972, TLI = 0.954, RMSEA = 0.073).

#### 2.2.3. Perceived Stress

The Chinese version of the Perceived Stress Scale (CPSS; Shi et al., 2019) was used to measure perceived stress. This 14-item scale comprises two dimensions: feeling out of control and tension. The feeling out of control dimension reflects difficulties in managing stress related to upcoming events (7 items, e.g., “feeling that one cannot control annoying emotions in life”). The feeling tension dimension captures heightened stress about forthcoming events (7 items, e.g., “feeling anxious and pressured”). Participants rated their agreement with each statement on a 5-point scale, ranging from 1 (“never”) to 5 (“always”). A higher mean score indicates a greater level of perceived stress. The validity and reliability of the CPSS have been confirmed among Chinese college students (Gong et al., 2021). The Cronbach’s α coefficient in the current study is 0.83. Confirmatory factor analysis indicated good structural validity (CFI = 0.925, TLI = 0.907, RMSEA = 0.071).

#### 2.2.4. AI Anxiety

This study used the AI Anxiety Scale developed by Y. Y. Wang and Wang (2022) to assess participants’ AI anxiety. This 21-item scale comprises four factors, including AI learning anxiety (8 items, e.g., “Taking a class about the development of AI techniques/products makes me anxious”), job replacement anxiety (6 items, e.g., “I am afraid that an AI technique/product may replace humans”), sociotechnical blindness (4 items, e.g., “I am afraid that an AI technique/product may be misused”), and AI configuration anxiety (3 items, e.g., “I don’t know why, but humanoid AI techniques/products scare me”). Responses were recorded on a 7-point Likert scale, with higher average scores indicating greater AI anxiety. The validity and reliability of this scale have been validated among Chinese faculty members and graduate students (Pan et al., 2025). In this study, Cronbach’s α coefficient is 0.96. Confirmatory factor analysis indicated good structural validity (CFI = 0.956, TLI = 0.948, RMSEA = 0.077).

#### 2.2.5. Intolerance of Uncertainty

Intolerance of uncertainty was assessed using the Intolerance of Uncertainty Scale-Short Form (IUS-SF; Y. J. Zhang et al., 2017), consisting of 12 items. The scale includes two factors: inhibitory anxiety and prospective anxiety. Inhibitory anxiety describes uncertainty inhibiting actions or experiences (5 items, e.g., “When it’s time to act, uncertainty paralyses me”). Prospective anxiety reflects anxiety about future uncertain events (7 items, e.g., “Unforeseen events upset me greatly”). Each item was rated from 1 (“not at all characteristic of me”) to 5 (“entirely characteristic of me”). Higher average scores indicate greater intolerance of uncertainty. The scale has been validated in Chinese university students with good reliability and validity (Shen et al., 2024). Cronbach’s α coefficient is 0.89 in this study. Confirmatory factor analysis indicated good structural validity (CFI = 0.956,TLI = 0.939, RMSEA = 0.073).

#### 2.2.6. Control Variables

The following demographic variables were included as control variables in the analysis: age, gender (1 = *male*, 2 = *female*), grade (1 = *first-year master’s*, 2 = *second-year master’s*, 3 = *third-year master’s*, 4 = *first-year doctoral*, 5 = *second-year doctoral*, 6 = *third-year doctoral*, 7 = *fourth-year doctoral*), monthly household income (1 = *below 1000 ¥*; 2 = *1001–3000 ¥*; 3 = *3001–5000 ¥*; 4 = *5001–10,000 ¥*; 5 = *10,001–20,000 ¥*; 6 = *20,001–40,000 ¥*; 7 = *above 40,000 ¥*), and address (1 = *urban*, 2 = *rural*).

### 2.3. Statistical Analysis

Statistical analyses were conducted using SPSS 27.0, Mplus 8.3, and R 4.4.3 for different analytical objectives. Preliminary analyses in SPSS included the Harman single-factor test for common method bias (Zhou & Long, 2004). The variance explained by the first factor was below 40%, indicating no common method bias. Descriptive statistics and correlation analyses were then computed. To examine conditional indirect effects, we used the PROCESS macro for SPSS (Model 89; Hayes, 2017), with simple slope analyses probing conditional effects at high and low levels of intolerance of uncertainty. Statistical significance was evaluated using bias-corrected bootstrapping with 5000 resamples, while effects were considered significant if the 95% confidence interval (CI) did not include zero. Additionally, confirmatory factor analysis (CFA) was conducted in Mplus to assess the discriminant validity of the main study variables. In R, discriminant validity was further assessed using the Heterotrait–Monotrait (HTMT) ratio, and regression diagnostics were conducted to check residual normality, heteroskedasticity, multicollinearity, and outliers.

## 3. Results

### 3.1. Check for Common Method Bias

Given the exclusive use of self-report measures in this study, the Harman single-factor test was used to examine the potential common method bias. The results showed that the eigenvalues of 10 factors were more than 1, and the variance explained by the first factor was 27.39%, substantially below the recommended threshold of 40%. Therefore, there was no significant common method bias in this study.

### 3.2. Confirmatory Factor Analysis and Discriminant Validity

To examine the discriminant validity of the main study variables, CFA was conducted on self-concept clarity, intentional self-regulation, perceived stress, AI anxiety, and intolerance of uncertainty. The factor structures of the alternative models were primarily guided by the correlations among the variables (see Table 1). For instance, in the four-factor model, perceived stress and intolerance of uncertainty were combined into a single factor due to their high correlation (*r* = 0.61). In the three-factor and two-factor models, additional variables with high intercorrelations were similarly consolidated. The results indicated that the five-factor model demonstrated significantly better fit indices (CFI = 0.919, TLI = 0.913, RMSEA = 0.043, SRMR = 0.084) compared to the four alternative models (see Table 2). This suggests that treating the five variables as distinct constructs provides the relatively optimal model fit. Furthermore, the HTMT ratio was used to assess the discriminant validity among the constructs. As shown in Table 3, all HTMT values were below the threshold of 0.85, suggesting good discriminant validity between the variables (Henseler et al., 2015).

### 3.3. Descriptive Statistics and Correlation Analysis

Table 1 presents the means, standard deviations, skewness, kurtosis, and Pearson’s correlation coefficients for self-concept clarity, intentional self-regulation, perceived stress, AI anxiety, and intolerance of uncertainty. The absolute values of skewness and kurtosis for the key variables are all below the common cutoffs (|skewness| < 2 and |kurtosis| < 7), suggesting a normal distribution of the sample on these variables. The correlation analysis showed that self-concept clarity is negatively correlated with perceived stress, AI anxiety, and intolerance of uncertainty, and positively associated with intentional self-regulation (*p* < 0.001). Intentional self-regulation is negatively correlated with perceived stress, AI anxiety, and intolerance of uncertainty (*p* < 0.001). Perceived stress is positively associated with AI anxiety and intolerance of uncertainty (*p* < 0.001). AI anxiety is positively correlated with intolerance of uncertainty (*p* < 0.001). These interrelations among the key variables provided support for the subsequent analyses.

### 3.4. The Moderated Chain Mediation Model Test

**Figure 2:**
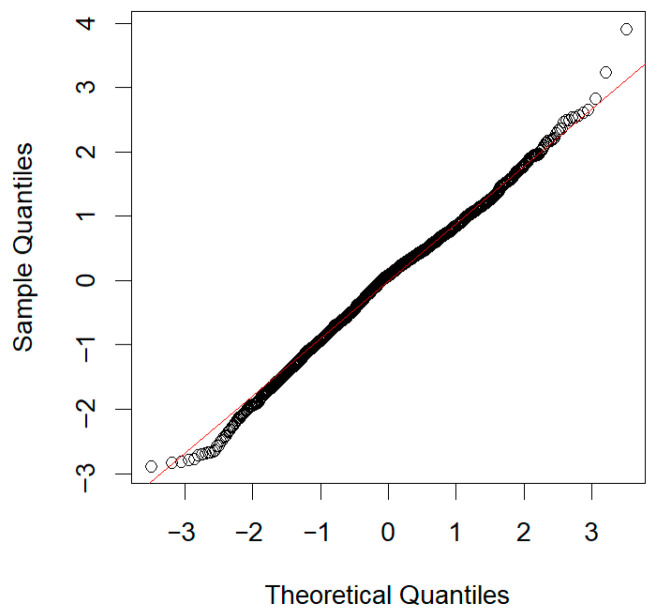
Normal Q-Q plot. Note. This plot assesses the normality of the data distribution. The circles represent the observed quantiles of the sample data against the theoretical quantiles expected under a normal distribution. The red line indicates the reference line of a perfect normal distribution. Deviation of the circles from the red line suggests departures from normality.

To ensure the robustness of the research findings, a comprehensive set of assumption checks for the regression model was conducted in R, including examinations of residual normality, heteroskedasticity, multicollinearity, and outlier diagnostics. First, the Shapiro–Wilk test for residual normality was statistically significant (W = 0.9953, *p* < 0.05), leading to the rejection of the null hypothesis of normality. However, the skewness (−0.158) and kurtosis (3.271) of the residuals were close to the theoretical values for a normal distribution, and the Q–Q plot (see Figure 2) showed that the residuals largely followed the reference line. This suggests that the departure from normality was methodologically acceptable. Second, the Breusch–Pagan test indicated the presence of heteroskedasticity (*p* < 0.05). Third, the collinearity statistics showed that the VIF values for self-concept clarity, intentional self-regulation, perceived stress, and intolerance of uncertainty were 1.75, 1.22, 2.07, and 1.82, respectively, which indicated a low possibility of multicollinearity (VIF < 5). Fourth, outlier diagnostics identified 8 outliers (0.37%), 157 high-leverage points (7.22%), and 136 influential points (6.25%) among the 2176 observations.

**Figure 3:**
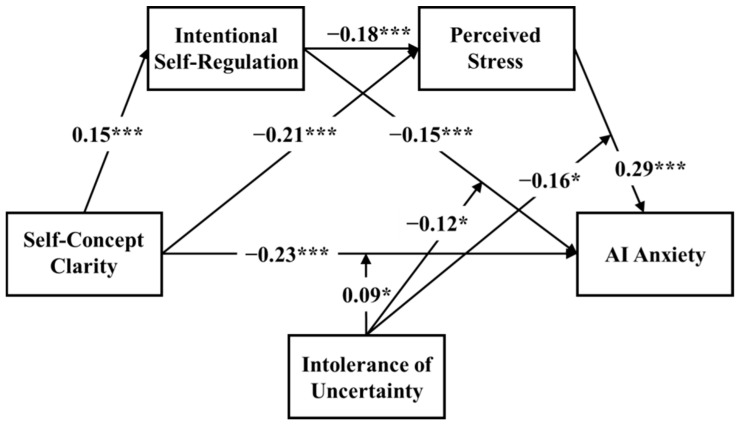
The moderated mediation model; * *p* < 0.1, *** *p* < 0.01.

To address the effects of heteroskedasticity and outliers, all variables were mean-centered, and statistical inference was performed using heteroskedasticity-robust standard errors (HC3). The results showed that HC3 standard errors were on average 14% larger than the ordinary least squares (OLS) standard errors, with the greatest increase observed for the interaction terms (ratio up to 1.40). After applying HC3 standard errors, the significance of three key interaction terms was attenuated from *p* < 0.05 to marginal significance (*p* = 0.076–0.091), while the significance of the other main effects remained unchanged. In summary, although the data exhibited some degree of heteroskedasticity and outliers, the main conclusions of this study remained robust under the more conservative HC3 standard error correction. For conciseness, only the results based on HC3 standard errors are reported in Table 4 and Figure 3.

Then, the moderated mediation analysis was conducted to examine the conditional indirect effects. As shown in Table 5, the indices of moderated mediation for all three indirect pathways were non-significant. This indicates that while intolerance of uncertainty demonstrated marginally significant interaction effects on the three direct paths, it did not translate into statistically significant alterations in the overall indirect effects of the mediation pathways. Consequently, the following discussion focuses primarily on the interaction effects observed in the direct paths.

**Figure 4:**
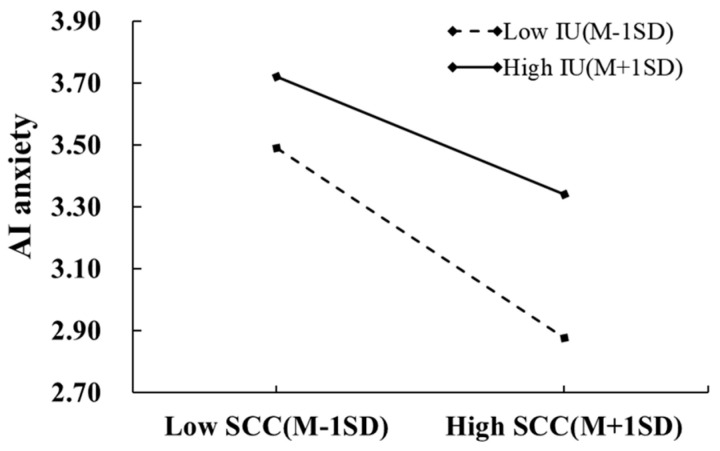
The moderating effect of intolerance of uncertainty between self-concept clarity and AI anxiety.

The interaction between self-concept clarity and intolerance of uncertainty was a marginally significant predictor of AI anxiety (*β* = 0.09, *p* < 0.1), suggesting that intolerance of uncertainty moderated the influence of self-concept clarity on AI anxiety. To probe these interaction effects, simple slope analysis was then conducted by evaluating the conditional effects at high and low levels of intolerance of uncertainty (plus or minus one standard deviation; Figure 4). The results showed that self-concept clarity has a significant impact on AI anxiety, regardless of high or low levels of intolerance of uncertainty. However, compared to graduate students with high intolerance of uncertainty (simple slope = −0.18, *p* < 0.001), self-concept clarity has a stronger effect on AI anxiety among individuals with lower intolerance of uncertainty (simple slope = −0.28, *p* < 0.001).

**Figure 5:**
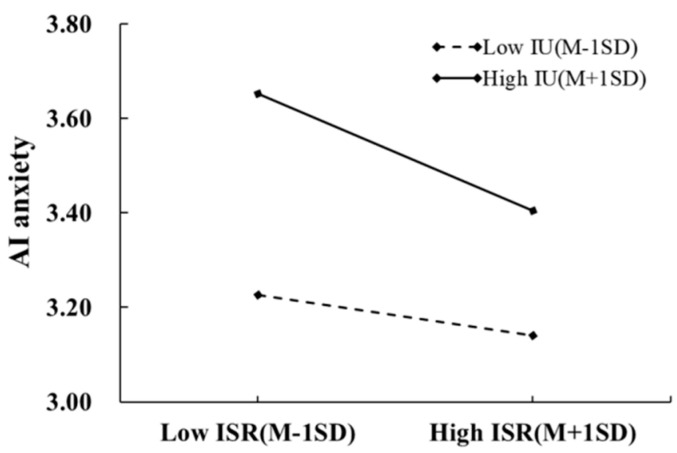
The moderating effect of intolerance of uncertainty between intentional self-regulation and AI anxiety.

The interaction between intentional self-regulation and intolerance of uncertainty has a marginally significant predictive effect on AI anxiety (*β* = −0.12, *p* < 0.1), indicating that intolerance of uncertainty moderated the relationship between intentional self-regulation and AI anxiety. As shown in Figure 5, simple slope analysis showed that under conditions of high intolerance of uncertainty, the negative predictive effect of intentional self-regulation on AI anxiety was significant (simple slope = −0.23, *p* < 0.001). However, under conditions of low intolerance of uncertainty, the effect of intentional self-regulation on AI anxiety was not significant (simple slope = −0.08, *p* > 0.05).

**Figure 6:**
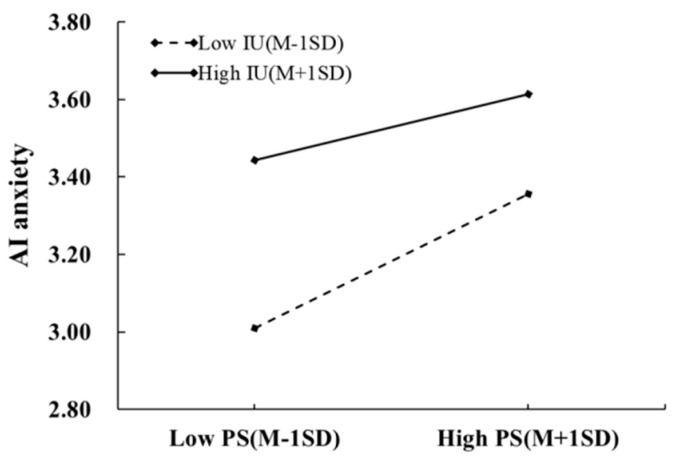
The moderating effect of intolerance of uncertainty between perceived stress and AI anxiety.

Furthermore, the interaction between perceived stress and intolerance of uncertainty was a marginally significant predictor of AI anxiety (*β* = −0.16, *p* < 0.1), indicating that intolerance of uncertainty moderated the impact of perceived stress on AI anxiety. As shown in Figure 6, simple slope analysis showed that the positive effect of perceived stress on AI anxiety was weaker among individuals with high intolerance of uncertainty (simple slope = 0.19, *p* < 0.01) compared to those with low intolerance of uncertainty (simple slope = 0.39, *p* < 0.001).

Additionally, methodological checks indicated that AI anxiety scores in the group of high intolerance of uncertainty (*M* = 3.78, skewness = −0.24, kurtosis = −0.27, maximum = 6.57 on a 1–7 scale) were neither near the scale ceiling nor negatively skewed. Therefore, a ceiling effect is unlikely to account for the moderating pattern observed in this study.

## 4. Discussion

Despite the fact that self-concept clarity is closely associated with mental health, few studies have explored whether and how self-concept clarity affects AI anxiety. This study utilized a cross-sectional design to elucidate the relationship between self-concept clarity and AI anxiety among graduate students, and further reveal the mediating roles of intentional self-regulation and perceived stress, as well as the moderating role of intolerance of uncertainty. Results demonstrated that self-concept clarity not only reduced AI anxiety directly, but also exerted indirect effects through independent and chained mediation pathways of intentional self-regulation and perceived stress. Furthermore, intolerance of uncertainty moderated the links from self-concept clarity, intentional self-regulation, and perceived stress to AI anxiety. These findings suggest that self-concept clarity serves as a significant protective factor against AI anxiety in graduate students by improving intentional self-regulation and mitigating stress perception, while highlighting intolerance of uncertainty as a risk factor that exacerbates AI anxiety.

The negative association between self-concept clarity and AI anxiety provides empirical support for Hypothesis 1. This finding is consistent with previous studies showing that individuals with low self-concept clarity are more susceptible to negative emotions provoked by outside events (Kusec et al., 2016; Van Dijk et al., 2014). From a theoretical perspective, these results substantiate the cognitive theory of anxiety, which posits that how individuals perceive themselves and organize self-knowledge information is the foundation for generating anxiety (Orr & Moscovitch, 2015). Individuals with low self-concept clarity often feel confused about “what kind of person am I”, and therefore are more likely to be troubled by negative emotions such as anxiety (Wu et al., 2017). The confused self-concept will raise concerns among graduate students about their professional or academic competitiveness, particularly in a rapidly evolving technological landscape, where they might perceive AI as a “competitor” that threatens their self-worth (Brougham & Haar, 2018). Conversely, clear and stable self-concept provides them with a stable self-referential framework (Jiang et al., 2020), enabling them to effectively inhibit catastrophizing cognition of technological challenges from AI tools and adopt proactive coping strategies (Bechtoldt et al., 2010; Kusec et al., 2016).

The significant mediation effect of intentional self-regulation and perceived stress in the relationship between self-concept clarity and AI anxiety support Hypothesis 2. The finding regarding intentional self-regulation indicates that high levels of self-concept clarity enables one to identify and prioritize self-initiated and valued goals, thus facilitating one’s self-regulation in long-term goal pursuit (Higgins, 1996; Light, 2017). Empirical evidence on goal striving and goal pursuit supports this assumption (Jiang et al., 2023; Wong & Vallacher, 2018). For instance, Jiang et al. (2023) suggest that when people feel confused about themselves, they will dismiss distal, larger-valued goals and advance proximal, smaller-satisfying goals. This finding also aligns with Gross’s (2015) process model of emotion regulation, particularly through the acceptance of AI-related benefits rather than emotional avoidance when confronting AI-related stressors (Cengiz & Peker, 2025).

The mediating role of perceived stress indicated that self-concept clarity can help alleviate AI anxiety by reducing individuals’ perceived stress. According to self-affirmation theory (Steele, 1988), clear self-representations facilitate self-affirmation, which enhances cognitive control and reduces excessive processing of threatening information, thereby lowering perceived stress levels (Ferrer & Cohen, 2019; Finley et al., 2018; Harris et al., 2017). Perceived stress further influences individuals’ reactions toward AI. Under conditions of elevated perceived stress, individuals tend to exhibit negative biases, focusing on the potential threats of AI rather than its opportunities—a maladaptive cognitive pattern that exacerbates anxiety (Burns & D’Zurilla, 1999; Hankin et al., 2016). Conversely, reduced perceived stress facilitates greater cognitive flexibility, enabling individuals to reinterpret AI-related uncertainties as manageable challenges rather than existential threats (Knauft et al., 2021; Liston et al., 2009), and to actively seek problem-solving strategies instead of rumination, thereby disrupting the exacerbation of anxiety (Kashdan & Rottenberg, 2010).

The chain mediation through intentional self-regulation and perceived stress provides support for Hypothesis 3. This finding is consistent with conservation of resources theory (Hobfoll, 1989). Studies consistently show that intentional self-regulation can effectively utilize and modulate available resources to mitigate perceived stress (Freund & Baltes, 2002; Vasquez et al., 2016). This stress-reduction effect further decreases anxiety and cognitive load, freeing cognitive resources for more adaptive responses to environmental changes (Huang et al., 2024). Building upon this established research, this study further extends the model of AI anxiety formation, proposing that it may be a synergistic product of identity crisis and technological alienation pressure. Specifically, when individuals fail to maintain a coherent self-narrative amid technological disruption, the depletion of self-regulation resources exacerbates perceived stress, leading to more anxious reactions toward AI. In conclusion, intentional self-regulation and perceived stress may serve as crucial explanatory mechanisms through which self-concept clarity buffers against AI anxiety, which underscore the clinical potential of self-concept and stress interventions for mitigating AI anxiety.

The moderating results of intolerance of uncertainty showed that individuals with higher intolerance of uncertainty exhibited higher AI anxiety compared to their lower counterparts, which partially supported Hypothesis 4. Specifically, for individuals with high intolerance of uncertainty, the negative impact of self-concept clarity on AI anxiety was weakened. Indeed, individuals with high intolerance of uncertainty tend to hold negative self-efficacy beliefs and pessimistic outcome expectations regarding problem-solving (Z. Li et al., 2015; Ouellet et al., 2019). As a result, they may be more likely to perceive AI-related uncertainties as potential threats (Fu et al., 2024). This cognitive bias amplifies anxiety toward unknown risks, thereby weakening the buffering effect of self-concept clarity on AI anxiety.

Moreover, intolerance of uncertainty negatively moderated the effect of intentional self-regulation on AI anxiety. Specifically, intentional self-regulation significantly reduced AI anxiety among individuals with high intolerance of uncertainty, whereas this effect was insignificant in the groups of low intolerance of uncertainty. A possible explanation is that individuals with high intolerance of uncertainty tend to take actions to reduce uncertainty (Alschuler & Beier, 2015), as their hypersensitivity to ambiguity compels them to seek resolution strategies to regain a sense of control (Freeston et al., 1994). In contrast, individuals with low intolerance of uncertainty rendered regulatory efforts less critical for maintaining emotional equilibrium. This result underscores the compensatory role of intentional self-regulation in coping with cognitive vulnerabilities.

Furthermore, intolerance of uncertainty weakened the relationship between perceived stress and AI anxiety, contradicting our initial projections. First, we ruled out the possibility that this result stemmed from a measurement ceiling effect. A possible explanation is that individuals with high intolerance of uncertainty are more sensitive to stress and may actively alleviate anxiety through thought suppression, mental preparation, or self-regulation strategies (Guitart-Masip et al., 2023; Sahib et al., 2024). Conversely, individuals with low intolerance of uncertainty may lack such buffering mechanisms and permit a more direct stress-anxiety translation. This counterintuitive pattern suggests that the impact of perceived stress on AI anxiety can be complexly influenced by emotion regulation styles and coping strategies. An alternative plausible explanation is that individuals with high intolerance of uncertainty (*M* = 3.78) exhibit a significantly higher baseline anxiety level compared to those with low intolerance of uncertainty (*M* = 2.39; *t* = 15.35, *p* < 0.001). Research shows that individuals with high intolerance of uncertainty chronically interpret ambiguous situations as threatening, maintaining a sustained state of chronic anxiety (Brosschot et al., 2016; Koerner & Dugas, 2008). These individuals, therefore, establish a relatively high and stable anxiety baseline, rendering short-term fluctuations in perceived stress less impactful on overall anxiety levels. Conversely, the lower baseline anxiety of individuals with low intolerance of uncertainty allows variations in perceived stress to directly map onto changes in AI anxiety, thereby demonstrating a stronger predictive relationship.

### 4.1. Limitations and Future Research

While the present study contributes novel insights into the psychological mechanisms linking self-concept clarity to AI anxiety through a moderated chain mediation model, several limitations must be acknowledged when interpreting the findings. Firstly, the sample was limited to master’s and doctoral students in China, which limit the generalizability of current results. Future research should expand the sample to diverse age groups, occupations, and cultural backgrounds, to assess the broader applicability of our proposed model. Secondly, the cross-sectional design of this study only allows for the examination of correlations between variables rather than establishing causal relationships. Maxwell et al. (2011) have pointed that cross-sectional estimates of direct and indirect effects are often severely biased and can be either larger or smaller than their true longitudinal counterparts. Thus, the interpretive claims regarding this study’s findings must be viewed as tentative and hypothetical. Future research employing longitudinal or experimental designs is needed, which can track the development of AI anxiety over time and establish the temporal sequence of predictors, providing stronger evidence for the proposed mechanisms. Thirdly, the reliance on self-report measures may be subject to biases such as social desirability and recall biases. Future research could benefit from incorporating complementary data collection methods, such as behavioral data, physiological measurements, and experience sampling, to provide more objective and comprehensive insights into AI anxiety and its predictors. Finally, within the AI anxiety construct, the second-order CFA revealed that the factor loading of the AI learning anxiety dimension (0.576) on the global factor was relatively low. Therefore, using a total AI anxiety score may obscure the distinct effects associated with specific dimensions, particularly AI learning anxiety. Future research should examine the different dimensions of AI anxiety separately to obtain more nuanced insights.

### 4.2. Practical Implications

Based on the findings of this study, self-concept clarity serves as a key psychological resource for mitigating AI anxiety, and its underlying mechanism—a chain mediation through intentional self-regulation and perceived stress—offers clear intervention pathways for higher education and psychological practice. In the context of higher education, curriculum design and student support services could integrate activities that promote the development of self-concept, such as structured reflective exercises, narrative-building workshops, and value-clarification training, to strengthen students’ coherent sense of identity and career aspirations. Simultaneously, efforts should be made to cultivate students’ ability to employ intentional self-regulation strategies, including goal setting, resource optimization, and compensatory skills, enabling them to proactively adapt their learning pathways and career plans in response to technological change. At the level of psychological practice, counselors and educators can offer cognitive-behavioral therapy-informed interventions for high-anxiety groups, particularly students with intolerance of uncertainty, to help them reframe appraisals of uncertainty and develop emotion-regulation and stress-management techniques. Furthermore, disseminating knowledge about AI through group counseling or psychoeducational lectures may reduce its perceived threat and alleviate associated anxiety. These measures not only support individual psychological resilience in the age of AI but also provide a practical framework for building academically inclusive and mentally supportive environments in technologically evolving settings.

## 5. Conclusions

This study indicated the psychological mechanisms underlying the relationship between self-concept clarity and AI anxiety by examining the mediating roles of intentional self-regulation and perceived stress and the moderating role of intolerance of uncertainty. Individuals with higher self-concept clarity tended to exhibit more effective intentional self-regulation strategies, which reduced perceived stress and, in turn, alleviated AI anxiety. Intolerance of uncertainty exacerbated AI anxiety by moderating the associations of self-concept clarity, intentional self-regulation, and perceived stress, with AI anxiety. Theoretically, the present findings enrich the understanding of AI anxiety and extend the application of relevant psychological theories in the context of AI, offering critical insights into psychological adaptation in technologically driven environments. Practically, the findings offer a basis for developing targeted intervention strategies to reduce AI anxiety by enhancing self-concept clarity, cultivating intentional self-regulation, managing perceived stress, and addressing intolerance of uncertainty.

## Figures and Tables

**Table 1:** Descriptive analysis and bivariate correlations among variables (*N* = 2176).

Variables	1	2	3	4	5	6	7
1. Age	1						
2. Monthly household income	−0.00	1					
3. Self-concept clarity	0.17 ***	0.09 ***	1				
4. Intentional self-regulation	0.08 ***	0.10 ***	0.30 ***	1			
5. Perceived stress	−0.08 ***	−0.07 ***	−0.59 ***	−0.38 ***	1		
6. AI anxiety	−0.13 ***	−0.06 **	−0.44 ***	−0.22 ***	0.40 ***	1	
7. Intolerance of uncertainty	−0.11 ***	−0.02	−0.56 ***	−0.12 ***	0.61 ***	0.39 ***	1
*M*	23.60	3.92	4.72	3.95	2.81	3.30	2.94
*SD*	2.03	1.24	1.09	0.54	0.44	1.06	0.63
Skewness			−0.12	−0.70	−0.15	−0.23	−0.06
Kurtosis			−0.59	2.22	1.84	−0.22	0.56

Note. ** *p* < 0.01, *** *p* < 0.001. All scales used a Likert format: self-concept clarity (1–7), intentional self-regulation (1–5), perceived stress (1–5), AI anxiety (1–7), intolerance of uncertainty (1–5).

**Table 2:** Confirmatory factor analysis.

Model	χ^2^	df	CFI	TLI	RMSEA	SRMR
Five-factor model: SCC; ISR; PS; AIA; IU	10,697.72	2115	0.919	0.913	0.043	0.084
Four-factor model: PS + IU; SCC; ISR; AIA	37,330.78	2204	0.849	0.842	0.058	0.094
Three-factor model: SCC + PS + IU; ISR; AIA	29,885.21	2200	0.739	0.729	0.076	0.101
Two-factor model: SCC + PS + IU + AIA; ISR	47,956.08	2202	0.568	0.553	0.098	0.117
Single-factor model: SCC + ISR + PS + IU + AIA	59,120.79	2206	0.463	0.445	0.109	0.136

Note. SCC, self-concept clarity; ISR, intentional self-regulation; PS, perceived stress; AIA, AI anxiety; IU, intolerance of uncertainty.

**Table 3:** Heterotrait–monotrait (HTMT) ratio.

	SCC	ISR	PS	AIA	IU
SCC	-				
ISR	0.406				
PS	0.752	0.478			
AIA	0.515	0.191	0.363		
IU	0.614	0.280	0.632	0.395	-

Note. SCC, self-concept clarity; ISR, intentional self-regulation; PS, perceived stress; AIA, AI anxiety; IU, intolerance of uncertainty.

**Table 4:** Results of the moderated mediation model.

Model	M1(ISR)		M2(PS)		M3(AIA)	
Predictive Variable	*β*	*t*	*β*	*t*	*β*	*t*
Gender	0.13	4.56 ***	0.02	1.24	0.19	4.03 ***
Age	0.01	2.03 **	0.00	0.91	−0.00	−0.20
Grade	−0.00	−0.10	0.00	0.65	−0.04	−1.97 **
Monthly household income	0.03	2.50 **	−0.00	−0.52	−0.02	−0.92
Address	−0.01	−0.56	−0.01	−0.60	0.08	1.76 *
SCC	0.15	13.28 ***	−0.21	−25.72 ***	−0.23	−8.23 ***
ISR			−0.18	−10.07 ***	−0.15	−3.15 ***
PS					0.29	3.71 ***
IU					0.28	5.39 ***
SCC × IU					0.09	1.78 *
ISR × IU					−0.12	−1.74 *
PS × IU					−0.16	−1.69 *
R-sq	0.11		0.40		0.26	
F	41.33 ***		154.16 ***		60.38 ***	

Note. SCC, self-concept clarity; ISR, intentional self-regulation; PS, perceived stress; AIA, AI anxiety; IU, intolerance of uncertainty. For conciseness, only significance levels based on HC3 robust standard errors are shown. * *p* < 0.1, ** *p* < 0.05, *** *p* < 0.01.

**Table 5:** Conditional indirect effects.

Path	Low IU		Medium IU		High IU		Index of Moderated Mediation	
	Effect	95% CI	Effect	95% CI	Effect	95% CI	Effect	95% CI
SCC→ISR→AIA	−0.012	[−0.030, 0.004]	−0.023	[−0.038, −0.009]	−0.034	[−0.054, −0.013]	−0.018	[−0.035, 0.003]
SCC→PS→AIA	−0.085	[−0.124, −0.047]	−0.063	[−0.097, −0.030]	−0.042	[−0.084, 0.002]	0.034	[−0.004, 0.073]
SCC→ISR→PS→AIA	−0.011	[−0.016, −0.006]	−0.008	[−0.013, −0.004]	−0.005	[−0.011, 0.000]	0.004	[−0.000, 0.009]

Note. SCC, self-concept clarity; ISR, intentional self-regulation; PS, perceived stress; AIA, AI anxiety; IU, intolerance of uncertainty.

## Data Availability

Data is available upon reasonable request.
